# Molecular Pathogenesis and Peripheral Monitoring of Adult Fragile X-Associated Syndromes

**DOI:** 10.3390/ijms22168368

**Published:** 2021-08-04

**Authors:** Luis M. Valor, Jorge C. Morales, Irati Hervás-Corpión, Rosario Marín

**Affiliations:** 1 Instituto de Investigación Sanitaria y Biomédica de Alicante (ISABIAL), 03010 Alicante, Spain; 2 Laboratorio de Apoyo a la Investigación, Hospital General Universitario de Alicante, Av. Pintor Baeza 12, 03010 Alicante, Spain; 3 Instituto de Investigación e Innovación Biomédica de Cádiz (INiBICA), 11009 Cádiz, Spain; jorgcmorales@hotmail.com (J.C.M.); ihervas91@gmail.com (I.H.-C.); charoma29i@yahoo.com (R.M.); 4 Unidad de Investigación, Hospital Universitario Puerta del Mar, Av. Ana de Viya 21, 11009 Cádiz, Spain; 5 Unidad de Genética, Hospital Universitario Puerta del Mar, Av. Ana de Viya 21, 11009 Cádiz, Spain

**Keywords:** FXTAS, FXPOI, FXAND, premutation, blood, biomarker, FMR1, FMRP, endocrine, mitochondria, miRNA, transcription, GABA, telomere

## Abstract

Abnormal trinucleotide expansions cause rare disorders that compromise quality of life and, in some cases, lifespan. In particular, the expansions of the CGG-repeats stretch at the 5’-UTR of the Fragile X Mental Retardation 1 (*FMR1*) gene have pleiotropic effects that lead to a variety of Fragile X-associated syndromes: the neurodevelopmental Fragile X syndrome (FXS) in children, the late-onset neurodegenerative disorder Fragile X-associated tremor-ataxia syndrome (FXTAS) that mainly affects adult men, the Fragile X-associated primary ovarian insufficiency (FXPOI) in adult women, and a variety of psychiatric and affective disorders that are under the term of Fragile X-associated neuropsychiatric disorders (FXAND). In this review, we will describe the pathological mechanisms of the adult “gain-of-function” syndromes that are mainly caused by the toxic actions of CGG RNA and FMRpolyG peptide. There have been intensive attempts to identify reliable peripheral biomarkers to assess disease progression and onset of specific pathological traits. Mitochondrial dysfunction, altered miRNA expression, endocrine system failure, and impairment of the GABAergic transmission are some of the affectations that are susceptible to be tracked using peripheral blood for monitoring of the motor, cognitive, psychiatric and reproductive impairment of the CGG-expansion carriers. We provided some illustrative examples from our own cohort. Understanding the association between molecular pathogenesis and biomarkers dynamics will improve effective prognosis and clinical management of CGG-expansion carriers.

## 1. The Multiple Faces of FMR1 in Pathology

### 1.1. Brief Description of the Fragile X-Associated Syndromes

The Fragile X Mental Retardation 1 (*FMR1*) gene is located on the X chromosome and encodes the polysome-associated RNA-binding protein FMRP, which plays key roles in neuronal development and synaptic plasticity through the regulation of mRNAs at the level of traffic, stability, splicing and both somatic and presynaptic translation [1]. The 5′-UTR of the *FMR1* gene contains a stretch of CGG repeats that causes a series of pathological conditions when exceeding 54 repeats, although there is increasing evidence of an association between the “gray zone”, ranging from 45 up to 54 triplet copies (which forms an unstable *FMR1* allele that can be expanded in successive generations [2]) with atypical parkinsonism [3]. The silencing of the *FMR1* gene occurs over 200 CGG repeats (the so-called full mutation, FM), resulting in severe diminished FMRP expression during development and in consequence the onset of the Fragile X syndrome (FXS, OMIM #300624). FXS is the most common cause of monogenic intellectual disability, with a high incidence of autistic features, hyperactivity behaviour and seizures, and usually accompanied by macroorchidism and distinct facial features [4].

The so-called premutation (PM), whose carriers can exhibit diverse symptomatology comprising both neural and non-neural pathological traits with different degrees of severity and compromised well-being, is defined in the range of 55 and 200 repeats. The worldwide prevalence of the PM is approximately 1:300 in females and 1:850 in males [5]. The risk to develop FXTAS (OMIM #300623) is higher in men than in women: from 17% to 75% of the PM male carriers as age increases, and only from 8 to 16% in PM carrier women [6,7] who exhibit a diverse phenotype much milder than those observed in men, possibly due to the female inactivation of chromosome X [8]. FXTAS is a late onset neurodegenerative disorder characterized by intention tremor usually accompanied by slow movement and parkinsonism, which develop into cerebellar gait ataxia and dystonia as the disease progresses, and by cognitive deficits (e.g., short-term memory loss, executive functions impairment, language capacity affectation), mood disorders and neuropsychiatric alterations, peripheral neuropathy, sleep disturbance and other signs [6,9,10]. The main radiological signs consist of an increased signal in brain white matter, especially in the middle cerebellar peduncles (MCP) in men [11]. MCP are the main afferent pathway to the cerebellum and are primarily composed by white fibers projected from the contralateral pontine nuclei as part of the cortico-ponto-cerebellar pathway that control motor tasks, planning and initiation of movements [12]. In addition, FXTAS patients show smaller volume in overall brain and particularly cerebellum related to PM carriers without diagnosis [13]. The presence of eosinophilic intranuclear ubiquitin-positive inclusions in brain, spinal cord and peripheral tissues is a hallmark of this disorder [14,15,16].

More prevalent in women is the development of the Fragile X-associated primary ovarian insufficiency (FXPOI, OMIM #311360): 24% of the PM women whereas the prevalence of POI in the general population is 1% [17]. This form is the most common inheritable ovarian dysfunction, which symptoms include irregular menstruation cycles, reduced fertility and early menopause onset [18]. More precisely, FXPOI involves ovarian hormonal dysfunction alongside with follicle depletion before the age of 40 years, having a direct impact on menstrual cycle regularity (that can lead to amenorrhea) and the ability to conceive, together with a variety of indirect consequences derived from chronic hypoestrogenism, such as early onset of osteoporosis and bone fracture, impaired vascular endothelial function, earlier onset of coronary heart disease and increased cardiovascular mortality, and higher risk of psychiatric symptomatology (e.g., anxiety, depression, etc.) than women with normal ovarian functionality [19,20,21,22,23,24].

This review is focused on the adult Fragile X-associated syndromes, starting with a brief description of the molecular pathogenic mechanisms caused by the CGG expansion, followed by a discussion about how some molecular and cellular alterations can be monitored at the peripheral level, mainly through the assessment of blood and derivatives: serum, plasma and peripheral blood mononuclear cells (PBMCs).

### 1.2. The Molecular Pathogenesis of Adult Fragile X-Associated Syndromes

**Figure 1:**
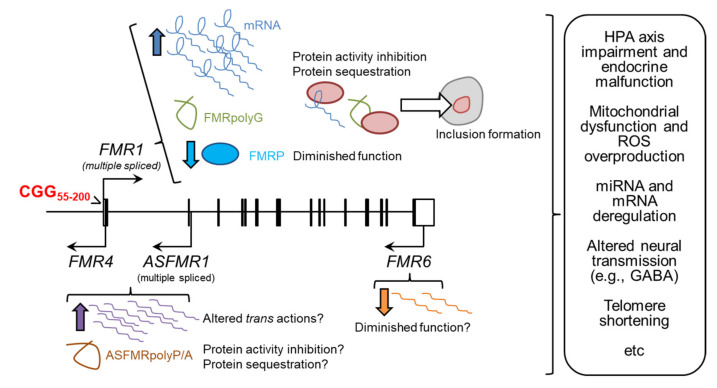
Summary of the molecular mechanisms proposed for the PM of the *FMR1* gene. Arrows indicate increases/decreases of RNA (*FMR1* and the long non-coding RNAs *ASFMR1*/*FMR4* and *FMR6*) or protein (FMRP) in PM carriers compared to controls. Pink ovals represent free proteins that interact/are sequestered/are aggregated by the CGG hairpin of the *FMR1* mRNA and/or FMRpolyG peptide.

In FXS, the methylation of the FM together with the neighbouring CpG island of the *FMR1* promoter triggers the silencing of the *FMR1* gene, leading to the absence or a drastic reduction of FMRP during development [25]. In contrast, FXTAS and FXPOI are primarily gain-of-function disorders, as FM carriers do not develop these syndromes. As we will see later in the section “Is there a Fragile X spectrum disorder?”, PM carriers may exhibit some forms of FXS signs. The main actions of the PM are summarized below (Figure 1).

#### 1.2.1. RNA Toxicity

Adult Fragile X-associated syndromes were firstly proposed to be the result of RNA toxicity, based on the elevated levels of *FMR1* mRNA and its presence in the ubiquitin-positive inclusions across different cell types in PM carriers [26,27,28]. The mechanism for *FMR1* upregulation in PM carriers is not well understood, and only correlative evidence for an increase in the binding of epigenetic marks at the *FMR1* gene promoter has been found, as in the case of H3K9ac and H3K9me2 in PM infertile women [29]. As reported in locus-directed stable cell lines, CGG repeats in the PM size alone is not sufficient to increase the mRNA levels of a reporter gene [30], therefore additional regulatory mechanisms may exist. The preferential usage of particular sites for transcription start and 3′-polydenylation in the brains of PM carriers compared to controls [31] may be indicative of differences in the regulation of *FMR1* gene expression. Another possibility is that preceding downregulation of specific miRNAs (see section “miRNAs as potential biomarkers in Fragile X-associated syndromes”) may influence the decay of *FMR1* mRNA, as in the case of miR-221 in synaptosomal preparations from KI mouse model, and miR-139-5p in lymphocyte-derived cell lines from patients, both with demonstrated capability to interact with the 3′UTR of *FMR1* mRNA [32,33].

Besides *FMR1* mRNA, the intranuclear inclusions also contain several proteins, suggesting that their sequestration by the PM RNA (riboCGG) caused a titration effect that impaired the biological processes in which these proteins are involved. For a detailed discussion of the role of the sequestered proteins in the FXTAS, see [34,35]. Mass spectrometry analyses, validated in silico predictions and screens for modulators of neurodegeneration eye phenotype in Drosophila PM models have provided a comprehensive list of potential interacting partners with riboCGG that, not surprisingly, included several RNA-binding proteins [36,37,38,39,40,41]. The first reported proteomics approach determined the composition of inclusions isolated from frontal cortex of FXTAS patients by fluorescent-activated cell sorting (FACS) techniques, taking advantage of the particle characteristics of inclusions and ubiquitin labelling. This analysis revealed the presence of RNA-binding proteins (e.g., the heterogeneous nuclear ribonucleoprotein A2/B1 (hnRNP A2/B1) and the myotonic dystrophy-related muscleblind-like splicing regulator 1 (MBNL1)), but also histones (e.g., H2A, H2B, H4), intermediate filaments and microtubules (e.g., NEFL, laminA/C, tubulins), and myelin associated proteins (e.g., MBP, CNPase) [36]. The interaction of hnRNP A2/B1 was later confirmed using a riboCGG in the PM range as a bait for mouse cerebellar proteins, which appeared besides another RNA-binding protein, Pur-α [37]. However, a subsequent study claimed that most of these proteins bound preferentially to non-pathogenic short sizes of riboCGG, whereas other proteins that included the DiGeorge Syndrome Critical Region Gene 8 (DGCR8) seemed to bind exclusively to longer CGG repeats within the pathogenic range [39] (see the section “miRNAs as potential biomarkers in Fragile X-associated syndromes”). Lastly, analysis of frontal cortex inclusions from FXTAS patients (isolated thanks to their autofluorescent properties, avoiding any bias from immunolabelling) resulted in the identification of > 200 proteins although no one showed any predominant abundance: histones, ubiquitin, proteins involved in RNA-binding (e.g., hnRNA A1 and A3), RNA splicing (e.g., U2AF, SFPQ), and protein turnover (e.g., chaperones and proteasomal-ubiquitin members such as SUMO 2 and p62), DNA damage repair mediators (e.g., RAD50, RPA1, XRCC6), etc. Of these, only five proteins (SUMO2, p62, ubiquitin, Myeloid leukemia factor 2 (MLF2) and MBP) were especially enriched in FXTAS aggregates related to total protein and related to the proteomics profile of control samples [40]. Based on this observation, the authors of this work postulated that inclusions are accumulations of proteins ready for removal but instead they aggregate once the capacity of proteasomal degradation is exceeded [40].

#### 1.2.2. Production of FMRpolyG Peptides

In addition to RNA, a pathogenic role has been proposed for a cryptic polyglycine peptide, FMRpolyG, that is produced by a repeat associated non-AUG (RAN) translation process occurring at the 5′-expanded CGG region of *FMR1* [42,43]. First discovered in the *ATXN8* gene which is involved in the Spinocerebellar ataxia 8 (SCA8), the products of RAN translation have been detected in vivo for other genes containing repetitive nucleotides such as Huntington’s disease (HD), myotronic dystrophy type 1 and 2 (DM1, DM2), amyotrophic lateral sclerosis (ALS) and frontotemporal dementia (FTD) [44]. In the *FMR1* locus, RAN translation can generate three types of homopeptides depending on the translation initiation (FMRpolyR, FMRpolyG and FMRpolyA), being the most efficient produced and the most well characterized the FMRpolyG (reviewed in [35]). In addition, FMRpolyG is the only species that have been found in intranuclear aggregates in neural and non-neural tissues (e.g., kidney, heart, thyroid, adrenal gland) [45], and in the cytoplasm of mural granulosa cells [46]. More recently, a vascular phenotype has been observed in the brain of PM carriers linked to the detection of FMRpolyG/p62+ inclusions [47]. Evidence for FMRpolyG toxicity has been collected from Drosophila and mouse models expressing different variants of engineered FMRpolyG aimed at enhancing or precluding such toxicity [42,48]. However, a recent study suggested that the appearance of FMRpolyG in neuronal intranuclear inclusions is not sufficient per se to trigger a clear phenotype in an inducible mouse model [49]. This is in contrast with the results obtained in a cellular model specifically designed to express the FMRpolyG in the absence of a CGG mRNA-dependent phenotype, in which cell viability was compromised and the nuclear lamina architecture was disrupted [50]. The C-terminus of the FMRpolyG (consisted on 42 aas after the polyG stretch) seems to be fundamental to the peptide toxicity as binds to cellular proteins such as LAP2β, a lamina associated protein, leading to the disorganization of the nuclear lamina architecture [48] (see the section “Transcriptional dysregulation in Fragile X-associated syndromes”). Another proposed mechanism for FMRpolyG-mediated toxicity is the acceleration of the ubiquitin-proteasome system impairment, as observed in Drosophila and cellular preparations [51]. This is in apparent disagreement with the proteomics study described above indicating that the proportion of FMRpolyG in intranuclear inclusions was too low in comparison with the total amount of proteins, putting into question its relevance in FXTAS pathogenesis [40].

#### 1.2.3. The Potential Role of Long Non-Coding FMR1 Isoforms

Long non-coding RNAs (lncRNAs) are mainly transcribed by RNA polymerase II, and are often subjected to 5′-end capping, 3′-end polyadenylation and splicing as protein-coding mRNAs, and can play multiple roles in the regulation of transcription and splicing, translation, DNA replication and response to DNA damage through their interaction with RNA-binding proteins, transcription factors and regulatory protein complexes such as the polycomb repressive complex 2 (PRC2) [52]. This type of transcripts have been also described for the *FMR1* locus, starting with the identification of the antisense FMR1 transcript (*ASFMR1*) between 2.5 kb upstream and 10 kb downstream of the transcription start site (TSS) of the *FMR1* gene, encompassing exons 1 and 2, intron 1, and upstream regions [53]. This region also includes *FMR4*, a non-coding antisense RNA of 2.4 kb which expression is initiated at the 5′UTR of the *FMR1* gene [54] and can be considered one of the multiple spliced isoforms of *ASFMR1*. Two additional *FMR1*-derived lncRNAs have been detected by using a combination of rapid amplification of cDNA ends (RACE) and next-generation sequencing (NGS) [55]: *FMR5*, a sense-oriented and unspliced 800 nt transcript in which 5′-end starts approximately 1 kb upstream of the TSS of *FMR1*, and *FMR6*, a 600 nt antisense transcript that originates in the 3′UTR of *FMR1* gene and ends in exon 15, sharing the same splice junction with *FMR1* mRNA.

The potential relevance of *FMR1*-derived lncRNAs in Fragile X-related syndromes is based upon the deregulation observed in PM carriers that can be associated with pathological traits, as further discussed in the section “The molecular outputs of the *FMR1* locus as biomarkers in pathology”. Expression of *ASFMR1* and *FMR4* is increased in the PM condition compared to controls, but negligible in FM condition in brain, peripheral blood leukocytes and lymphoblastoid cell lines [53,54,55]. *FMR5* and *FMR6* are also widely expressed across brain areas and peripheral blood samples, but whereas *FMR6* expression was reduced in PM and FM carriers related to controls in post-mortem brain tissue, *FMR5* levels did not show differences between the three groups of individuals [55]. In granulosa cells of PM women, the RNA levels of *FMR6* (but not those of *FMR4*) showed a significant negative linear correlation with the number of retrieved oocytes and a significant non-linear association with the number of CGG repeats (with the highest levels in women with mid-size CGG repeats) [56], indicating the *FMR6* may be involved in ovarian dysfunction. However, the pathogenic mechanisms underlying their contribution in disease are largely unknown, although there are clues indicating a *trans* action that is independent on *FMR1* regulation. RNA interference and over-expression assays in transient transfection experiments revealed that the expression of *FMR1* and *FMR4* is not influenced by each other [54]. *FMR4* has anti-apoptotic and cell proliferative actions in human cell lines and neural precursors [54,57] and may have a physiological function during neural development [58]. Manipulation of the *FMR4* levels in HEK293T cells leads to genome-wide changes in gene expression and DNA occupancy of H3K4me3 and K27me3, two epigenetic marks associated with active and silent genes respectively [57,58], indicating that *FMR4* is a *trans* regulator of gene expression and chromatin modulation. Based on these results, it is expected that the actions of *FMR1*-lncRNAs can be altered in PM cells.

In addition, *ASFMR1* and *FMR4* contains CCG repeats in the complementary strand that may be deleterious as well: in this regard, ASFMRpolyP and ASFMRpolyA peptides as a result of RAN translation of the *ASFMR1* transcript can be detected in FXTAS brains [59].

#### 1.2.4. Reduction of FMRP Activity

Elevation of *FMR1* mRNA has been detected concomitant to a modest reduction of FMRP in some (although not all) PM individuals [60,61], possibly as a homeostatic mechanism to minimize the deleterious actions of the mRNA [62]. Such reduction may act in combination with the gain-of-function component of the PM to exacerbate some aspects of the complex symptomatology exhibited by the carriers. More specifically, this contribution has been proposed to potentially explain mental traits as we will discuss below, due to the role of FMRP in synapse regulation [63]. Noticeably, PM carriers showing both *FMR1* mRNA increase and FMRP decrease may develop psychotic and bipolar disorder features that are extremely rare in FXTAS and FXS patients, probably due to a synergistic effect of the gain- and loss-of-function components of CGG expansion [64].

### 1.3. Is There a Fragile X Spectrum Disorder?

Some authors have suggested that all clinical manifestations associated with the PM of the *FMR1* gene constitute a spectrum of varying degrees of penetrance and severity of these clinical signs, in which the final diagnosis of FXTAS or FXPOI may represent extreme forms of cognitive and endocrine impairment that may be present in other PM carriers in a milder manner. Therefore, the motor, cognitive and reproductive impairments generally ascribed to FXTAS and FXPOI diagnoses do not follow a strict boundary between sexes. Meanwhile PM women who are asymptomatic for classical FXTAS may develop subtle motor and cognitive impairments that are suspicious to be cerebellar-dependent [65], PM men can develop intranuclear inclusions in brain as well as in testicular tissues, prominently in the Leydig cells that are responsible for secreting testosterone to maintain spermatogenesis [15,16,66]. A potential association of such inclusions with cases of infertility [66] resembles the situation of granulosa cells and POI in PM women. Moreover, there is also a major risk of macroorchidism in PM men, a clinical feature usually linked to FXS that has been correlated with lower verbal and intelligence quotient (IQ) in the examined individuals [67].

Thus, manifestation of specific traits can be largely dependent on unknown genetic interactions, and/or the degree of mosaicism between the gain- and loss-of-function components of the CGG expansion that may differ throughout neural and non-neural tissues. For instance, patients who presented both *FMR1* mRNA increase and FMRP decrease manifested a symptomatology resembling combined FXS and FXTAS [64], although the mosaicism of methylated and unmethylated FM alleles can also explain the coexistence of FXS and FXTAS-related diagnosis in the same individuals [64,68]. It has been documented that pediatric PM carriers may develop attention deficit hyperactivity disorder (ADHD), anxiety, autistic features, seizures and other psychiatric symptoms that are reminiscent of FXS but to a much lesser extent and prevalence compared to FXS patients [69]. Therefore, PM boys have higher risk to be diagnosed of autism spectrum disorder (ASD) or ADHD than age-matched PM girls, although in both groups the risk was higher than in controls, leading to the hypothesis that reduced protein levels of *FMR1* are responsible for the mild signs of the developmental and cognitive impairments observed in PM carriers that are characteristic of FXS patients [70,71].

However, the majority of PM children do not have psychiatric conditions but they are at higher risk than the general population to develop them in the mid-adulthood, independently of a FXTAS diagnosis. The term Fragile X-Associated Neuropsychiatric Disorders (FXAND) has been coined to group the diverse neuropsychiatric problems observed in PM carriers that precede the onset of characteristic FXTAS but recognizes a series of mental disturbances in those PM carriers that do not fall into the diagnosis of FXTAS and can be more common than expected in these individuals [72]: anxiety, high sensitivity to external stimuli, depression, ADHD, obsessive-compulsive disorder, chronic pain and fatigue, sleep disturbance, and drug abuse (probably as part of a self-medicating behaviour) [72,73]. This term also considers non-neurological symptoms, as in the case of autoimmune conditions that mainly appear in PM females (hypothyroidism, fibromyalgia, irritable bowel disease, etc.) [19].

## 2. Peripheral Blood as Source of Biomarkers for Adult Fragile X-Associated Syndromes

Due to the pleotropic actions of the *FMR1* PM which complex manifestations of symptoms is being increasingly recognized in the affected population [9], it is indispensable to monitor the health status of their carriers through the molecular assessment of biomarkers that should follow essential requisites of being cost-effective, easily quantifiable in accessible tissues or fluids, and provide a linear correlation with disease progression (or reversal in case of treatment) [74]. In this sense, blood-based biomarkers offer a low-invasive alternative to screen and monitor different organ functions and associated diseases in clinical practice. Accomplishment of such requisites can be challenging in the case of FXTAS, in which the affected nervous tissues are not accessible and the potential biomarkers depend on either the non-neural component of the disease or in the extravasation of metabolites and other molecules to the periphery that should take place in early stages. Thus, interrogation of the heterogeneous cellular fraction (focused on PBMCs) relies on the assumption that the molecular events occurring in the affected organs that trigger the manifested symptomatology in PM carriers can be shared, or at least have a correlative impact, on these cells. In this context, plasma and serum can be relatively homogeneous sources of biomarkers, through the measurement circulating cell-secreted proteins, metabolites, extracellular vesicles and other components for patient screening. Omics approaches can discover novel and unforeseen biomarkers through the description of altered patterns of blood cells RNAs [75,76] and plasma metabolites in PM carriers compared to controls [77,78,79], as we will discuss later.

However, interpretation of the results obtained in most of the associative studies conducted in PM carriers should be taken as preliminary in the absence of a validation in independent cohorts, due to the small number of recruited volunteers (usually consisted on few individuals to few dozens) that is characteristic of studies in rare disorders. This can be an important reason for the lack of significant differences between PM carriers with or without a diagnosis of FXTAS or FXPOI, unless we consider that molecular alterations precede the onset of the symptomatology required for a conclusive diagnosis.

### 2.1. The Molecular Outputs of the FMR1 Locus as Biomarkers in Fragile X-Associated Pathology

The most examined peripheral parameter as a putative biomarker of the vast array of Fragile X-related symptoms and alterations is the number of CGG repeats in blood cells. In this sense, the most well-established association using the number of CGG repeats is the non-linear correlation with FXPOI diagnosis in which PM carriers with medium size CGG tracks have higher risk for ovarian insufficiency, altered cycles and dizygotic twinning [17,80]. Quantification of the number of repeats assumes that CGG size is fairly stable in somatic cells across lifespan [81,82] leading to negligible (or at least correlative) differences between peripheral blood cells and the affected organ. However, instability cannot be entirely discarded [83] as this is commonplace in other trinucleotide repeats disorders [84,85,86]. In fact, somatic expansion rates in PM alleles have been found to be different across human and mouse organs, being high in testis and certain brain areas (e.g., amygdala and striatum) but relatively stable in lymphocytes [87]. Such difference may introduce discrepancies in the number of repeats analyzed from blood and tissues of the same individuals.

Since the realization that *FMR1* mRNA was upregulated in blood samples bearing the PM range compared to controls, concomitant with a reduction in the number of FMRP^+^ lymphocytes [60], and fueled by early indications of an association of these changes with cognitive deficits in certain PM carriers [88], it has been usual to implement the examination of mRNA and/or protein levels besides the number of CGG repeats as potential correlates of the development of specific symptoms and the severity of the general pathological phenotype. This is particularly interesting, because such associations allow inferring whether the gain- and/or loss-of-function components of the *FMR1* PM (corresponding to RNA toxicity and FMRP activity reduction, respectively) can explain the onset of specific signs. In this direction, there have been attempts to correlate CGG size and *FMR1* mRNA levels with IQ [89] and psychiatric symptoms [90,91]. Of special interest, some studies have tried to correlate these peripheral parameters with alterations in brain structure and brain activation patterns as measured using non-invasive imaging techniques. These approaches also permit the identification of the neural substrates responsible for the altered brain functions of clinical relevance for the PM carriers. A summary of these studies is shown in Table 1.

Apart from the canonical mRNA for *FMR1*, the non-coding transcripts generated from the *FMR1* locus have been also screened for clinical associations, based on previous observations showing increased expression in peripheral leukocytes [53,54]. For instance, small CGG expansion *FMR1* alleles (i.e., gray zone and lower PM sizes) play a significant role in the development of the parkinsonian-like phenotype that has been associated with elevated *FMR1* and *ASFMR1*/*FMR4* levels in PBMCs, in parallel to deregulation of mitochondrial genes [100]. Moreover, a spliced variant of *ASFMR1* with a 84 nt-deletion near the TSS was more elevated than the unspliced transcript in PM carriers compared to controls, and have been tentatively associated with FXTAS diagnosis [101]. Another highly similar splicing isoform that lacks 131 bp near the TSS (*ASFMR1*-Iso131bp) was also higher expressed in PM carriers independently of a FXTAS diagnosis compared to controls, with a trend to be correlated with tremor intensity as measured in a series of tasks involving postural and intentional tremor, postural sway, manual coordination, and reaction time [102]. Despite not showing differences between FXTAS and non-FXTAS individuals, the increase in the levels of *ASFMR1*-Iso131bp has been later ascribed to the acquisition FXTAS symptomatology during a longitudinal study [103].

To add more complexity to the potential contribution of *FMR1* RNAs in the etiology of neural and non-neural symptomatology in PM carriers, up to 49 *FMR1* alternative splicing isoforms have been detected in blood and different tissues (brain, muscle, testis and heart) thanks to single-molecule long-read sequencing that enabled the mapping of all possible splicing combinations in a single *FMR1* mRNA molecule [104,105]. An important fraction of these isoforms were increased, and in some cases exclusive, in the PM group [104,105], thus providing new candidates for peripheral exploration. According to the authors, the most notable isoforms were those named as Iso10 and Iso10b that, together with isoforms Iso4 and Iso4b, lack the nuclear export signal (NES) and the glycine-arginine-rich (RGG) motif involved with RNA binding [106]. As happened with *ASFMR1*-Iso131bp, these isoforms increased during the transition from disease-free stage to declaration of FXTAS in PM men [103], suggesting that they may be candidates as biomarkers of disease progression. In agreement with this conclusion, there was a significant association between increased expression of Iso4/4b with the decreased width of MCP only in those individuals that become diagnosed of FXTAS during this longitudinal study [103].

Finally, the *FMR1* locus contains additional features that have been examined as potential peripheral biomarkers. The absence of AGG interruptions in the CGG tracts, leading to uninterrupted CGG repeats that may predispose to germline expansion of low and medium size PM to produce FM during offspring transmission [107], have shown some correlation with neurological signs that deserves further confirmation [101]. Moreover, cortical white matter thickness and executive dysfunction have been associated with changes in the methylation patterns of CpG dinucleotides located in the first exon/intron boundary in the peripheral blood in PM women who were asymptomatic for FXTAS [108,109].

### 2.2. Endocrine Biomarkers in Premutation Carriers

In POI, measurements of serum levels of follicular stimulating (FSH) and anti-Müllerian (AMH) hormones, estradiol (E2) and inhibin B can be performed using standard protocols to assess the ovarian reserve [110]. A proteomic approach enlarged the list of possible candidates as biomarkers of POI with the identification of proteins with potential actions over reproductive functions (ceruloplasmin, complement C3, fibrinogen α, fibrinogen β, and sex hormone-binding globin SHBG) [111]. Although measurements of circulating endocrine hormones and other compounds can monitor the POI linked to CGG PM (FXPOI), they hardly discriminate between POI of different origin in the absence of a genetic test for *FMR1* CGG repeats. In other words, the hormone profiles found in FXPOI patients are not specific of the PM but, instead, they relate to reduced ovarian reserve.

The most well-established change in PM women is the elevation of circulating FSH levels, as it is one of the requisites for POI diagnosis, that has been shown to be independent on the menstrual cycle phase, regularity of menstrual cycles and oral contraception treatment [112,113,114,115], and can explain the decrease in the number of follicles and the reduction of the follicle phase and menstrual cycle length [112]. This phenomenon was reproduced in mouse models in which there was a loss of all types of follicles despite normal development of the ovary and normal establishment of the primordial follicle pool [116,117,118]. In these mice, elevation of FSH led to follicle depletion due to the activation of the primordial follicle pool and accelerated atresia of follicles, whereas diminished levels of luteinizing hormone (LH) levels reduced ovulation; thus, murine ovaries showed the downregulation of genes related to LH-induced ovulation including the LH receptor, together with a reduction in the phosphorylation of Akt and mTOR [117] that can be relevant molecular events, given the relevant roles of PI3K-Akt and mTOR signalling pathways in granulosa cells differentiation and survival, and growth and maturation of the ovary which dysfunctions can provoke POI [119,120]. In addition, it has been suggested that the follicular atresia can be due to the imbalance of granulosa cells cycle towards apoptosis provoked by the PM [121]. FSH is primarily secreted by the anterior pituitary gland that can contain FMR1-inclusions [66,122], indicating that the deleterious effects of the PM may perturb the hypothalamic-pituitary-adrenal axis; of interest, studies in a CGG-PM mouse model revealed that the formation of inclusions in the pituitary and adrenal gland can be an early event that precedes aggregation in most parts of the brain [123].

In PM women, other hormone alterations have also been detected, such as the reduction of AMH [118,124], inhibin A and B and progesterone P4 [112]. Of note, inhibin B exerts an inhibitory action over FSH secretion [125]. However, measurements of ovarian dysfunction (FSH levels) and pituitary-adrenal dysfunction (prolactin, cortisol and ACTH levels) were not correlated with scores in the FXTAS motor rating scale in PM women [91], suggesting that endocrine and motor alterations in PM carriers do not have a simple relationship. Finally, no association has been observed between CGG repeats in the normal range and reproductive parameters (including FSH and AMH levels) [126], but there are some indications regarding high levels of FSH in cases bearing intermediate CGG sizes (35–54 CGG repeats) and fecundity problems and amenorrhea [115], an observation that deserves further confirmation.

### 2.3. Mitochondrial Dysfunction and Overproduction of Reactive Oxygen Species in Premutation Carriers

The mitochondria are responsible for the cellular energy production through the oxidative phosphorylation process, play key roles in apoptosis induction and homeostasis maintenance. During ATP synthesis by oxidative phosphorylation, mitochondria produce reactive oxygen species (ROS) that are essential for different biological functions as cell cycle progression, immune response and homeostasis, as well as signalling pathways mediators during developmental processes like maturation and differentiation [127,128,129]. However, increases in the production of oxidant agents are related to DNA damage and cell death, and are associated with human pathologies such as cancer, cardiovascular disease and neurological disorders [78,130,131,132]. In the case of *FMR1*, the ablation of the gene in a knockout mouse model produced a transient increase in ROS production, NADH-oxidase activity and altered glutathione homeostasis concomitant to enhanced lipid peroxidation and protein carbonylation in brain extracts [133]. Primary hippocampal neurons from CGG-KI mice (with >150 CGG) also showed abnormal mitochondrial trafficking and bioenergetics [134]. Nonetheless, a more detailed description of alterations has been provided in cultured dermal fibroblasts from PM carriers, including decreased NAD- and FAD-linked oxygen uptake rates, uncoupling between electron transport and synthesis of ATP, decreased mitochondrial protein expression, increased oxidative/nitrative stress, higher NADH-dependent hydrogen peroxide production, Zn and Fe transport impairment, abnormal mitochondrial morphology and increased ROS production that usually worsen in FXTAS-declared patients and can be correlated with the number of CGG repeats [78,135,136,137,138,139]. Isolated peripheral lymphocytes also present aberrant mitochondria which can be correlated with clinical hallmarks of FXTAS [140,141] and postmortem brains of FXTAS patients mirrored the decrease in the levels of mature proteins such as Sodium/potassium-transporting ATPase subunit β (ATPB), Manganese superoxide dismutase (MnSOD) and frataxin found in skin fibroblasts [135,137]. Moreover, the integrity and copy number of mitochondrial DNA (mtDNA) can be influenced by ROS production in pathological processes such as cancer, cardiac conditions and neurodegeneration [142,143,144]. Meanwhile the deleterious effects of ROS on mitochondrial energy production may cause an increment of the mitochondria number per cell in an attempt to preserve homeostasis and the release of cell-free circulating mtDNA, ROS can also promote its degradation depending on the pathological condition [145,146]. In the case of PM carriers, mitochondrial perturbations were not apparently caused by a reduction in mitochondrial numbers as measured by citrate synthase activity or mtDNA copy number per cell [135,137,138,140]. However, other studies reported a decrease in mtDNA copy number and an increase in the rate of deletions in PM carriers compared to controls regardless of FXTAS manifestation [78,141]. It has been suggested that the decrease in mtDNA copy number per cell is more evident in tissues or cells directly relevant with the clinical manifestations of the disease. Thus, lower mtDNA copy number per cell was reported in specific brain areas related to disease progression (cerebellar vermis, dentate nucleus, parietal and temporal cortex) from asymptomatic PM carriers that were exacerbated in FXTAS-declared patients but failed to be significantly different in cultured dermal fibroblasts and in the brains of CGG-KI mice that did not exhibit tremors [147].

Mitochondrial dysfunction in *FMR1*-dependent ovarian insufficiency has been addressed by using female mice of the 130R strain bearing an insertion of expanded CGG repeats in the *Fmr1* gene. Whereas ROS production at the physiological range is necessary for oocyte maturation, ovarian stereoidogenesis, corpus luteal regulation and luteolysis [148], the 130R strain showed decreased primordial follicles in adult stages, histomorphometric evidence of follicle atresia, reduction of granulosa cell number in both growing and adult follicles, presence of ovarian cysts, and diminished fertility among other abnormalities [116,149]. Transmission electron microscopy analysis also revealed a population of abnormal mitochondria in appearance and structure, with evidence of mitochondrial gene expression downregulation, together with reduced mitochondrial mass [149]. Finally, mtDNA copy number was decreased in granulosa cells and metaphase II eggs of this strain, concomitant with reduced mitochondrial mass measured by flow cytometry [149].

This vast catalogue of mitochondrial alterations has been proposed to be caused by the interaction between FMRpolyG-containing aggregates and the mitochondrial membrane, which was concomitant to negative effects on membrane potential, ATP synthesis, assembly of respiratory chain supercomplexes composed by complexes I, III and IV, and gene expression of mitochondrial mRNAs, in the absence of altered mtDNA content in in vitro preparations [150]. This mechanism is plausible to occur in the ovary as well because FMRpolyG has been detected in granulosa cells with expanded CGG repeats [46]. In addition, it is also feasible that the supply of nuclear-encoded mitochondrial genes is compromised [151]. More recently, it has been described that expression of FMRpolyG in the HEK293 cell line can altered the content of nuclear-encoded miRNAs in whole cellular preparations but also in mitochondrial fractions [152], indicating that the altered biogenesis of miRNAs in CGG PM conditions (as we will discuss in brief) also affect mitochondrial functions. However, some miRNAs were enriched in the mitochondria but depleted in whole cell lysates, as in the case of miR-320a, which translocation into the HEK293 mitochondria was apparently increased in response to CGG PM conditions in an attempt to restore mitochondrial functions but failing to form the functional RISC complexes with Argonaute 2 (Ago2) required for regulation of mitochondrial transcripts [152].

**Figure A1:**
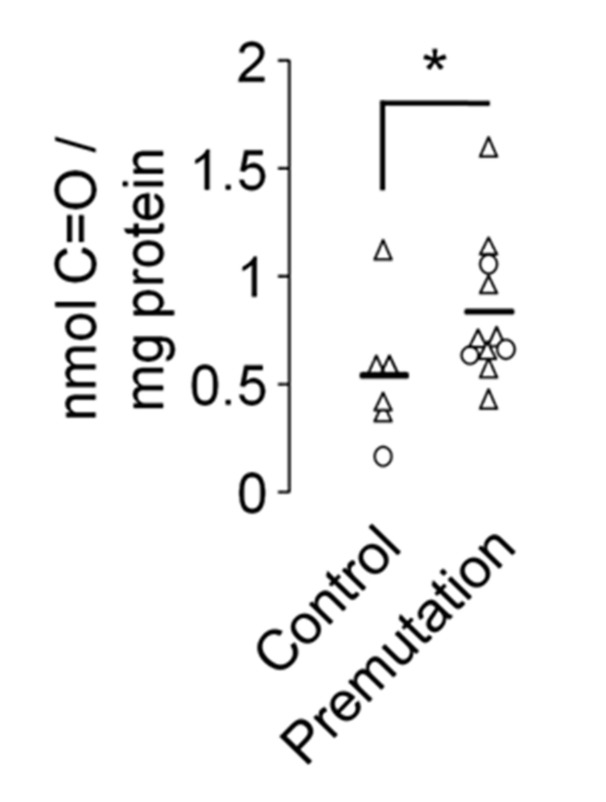
Significant increase of protein carbonylation in the plasma of premutation carriers. Triangles ( Δ), female samples; circles (O), male samples. The mean is indicated as a horizontal bar. *, *p* < 0.05, Mann-Whitney U-test.

In support of mitochondrial dysfunction as an early event in *FMR1*-CGG repeats disorders, it has been reported that the decreased expression of mitochondrial proteins in the brains of FXTAS patients required a lesser number of CGG repeats to be manifested than inclusion formation [137]. Morever, liver steatosis and linked mitochondrial dysfunction appeared in an inducible transgenic mouse model expressing expanded CGG in a timing when inclusions were not detected [153]. Based on these observations, together with the mitochondrial dysfunction observed in carriers without overt symptomatology, we can hypothesize that the effects of mitochondrial impairment and overproduction of ROS could be easily quantified at the peripheral level with sufficient anticipation to the onset of symptoms, providing reliable biomarkers in disease prognosis and therapeutic response. Noticeably, ROS production and mitochondrial function (e.g., citrate synthase activity, ATP production, etc.) in lymphocytes showed significant correlations with clinical outcomes in a cohort of PM women, such as diagnosis of FXTAS and FXTAS stage, diagnosis of FXPOI, number of CGG repeats, anxiety and full-scale IQ [79], suggesting that peripheral bioenergetics is directly linked to Fragile X-associated symptomatology. Metabolomics can go further in tracing the mitochondrial and oxidative perturbations in the plasma of PM carriers by identifying altered mitochondrial metabolic intermediates of the Krebs’ cycle [154], in addition to other metabolites that can be explained by oxidative stress-dependent damage to proteins and carbohydrates, indicative of interference with multiple metabolic pathways (e.g., lower catabolism of carnosine, hyperactivation of the polyol metabolic pathway, increased activity of urea cycle) and activation of NADH-dependent antioxidant mechanisms [155]. Another metabolomics study confirmed the increase in metabolites derived from oxidation of carbohydrates and proteins and the hyperactivation of the polyol pathway; in addition, cells from FXTAS-declared carriers (but not those derived from asymptomatic donors) showed significantly higher Tyr nitration of cytoskeletal proteins and increased malondialdehyde (MDA), a marker of lipid peroxidation [78]. Following these observations, protein carbonylation in plasma/serum can be an adequate proxy of ROS overproduction in PM carriers because of ROS release into the bloodstream (Figure A1 Appendix A). As this phenomenon is tightly linked to lipid peroxidation [156], a spectrophotometry method may be preferred to measure protein carbonylation that can be more easily standardised in clinical laboratories [157] than the more sensitive but less routinary high performance liquid chromatography (HPLC) for MDA measurements [158]. A proteomics approach was also applied to PBMC of PM carriers, reflecting alterations in components related with glycolysis and carbohydrate and pyruvate metabolism that were consistent with the Kreb’s cycle alterations found in plasma [79].

### 2.4. Inflammation in the Premutation Condition

Neuroinflammation, defined as the inflammatory response within the brain or spinal cord, is regarded as a relevant contributor of neurodegenerative processes. FXTAS seems not to be an exception as post-mortem putamen of patients showed signs of microglia activation, reflected by either the presence of dystrophic/senescent microglia or increased number of microglia (also detected in other brain areas) related to controls [159]. Moreover, post-mortem cerebellum contained increased levels of the proinflammatory IL-12 and TNFα, with a non-significant trend for IL-2, IL-8 and IL-10 [160]. At the peripheral level, it has been reported an increase in IL-10, an immunosuppressive cytokine that modulate glial activation to exert both protective and deleterious effects in neurodegenerative disorders [161], in the supernatants of PBMCs obtained from PM males that correlated with the number of CGG repeats but not with motor clinical scores [162]. In contrast, no changes were observed for IL-6 and IL-8 [162]. Overall, these few results suggest that exploration of circulating interleukins and cytokines profiles in Fragile X-associated syndromes can be worthy in the search of valuable biomarkers of disease progression.

### 2.5. miRNAs as Potential Biomarkers in Fragile X-Associated Syndromes

The binding of DGCR8 and its partner DROSHA with expanded CGG repeats and their presence in CGG RNA-containing aggregates indicated that miRNA processing was affected in PM carriers [39]. DGCR8, a double-stranded RNA binding protein, and DROSHA, a double-stranded RNA-specific ribonuclease, are part of the microprocessor complex that process the primary miRNAs (pri-miRNAs) into precursor miRNAs (pre-miRNAs) that are then exported to the cytoplasm for further processing by Dicer into mature miRNAs [163]. According to these roles, the partial sequestration of DGCR8 and DROSHA by CGG repeats led to reduced processing of pri-miRNA into pre-miRNA in cellular preparations [39], a phenomenon that explained the predominant downregulation of mature miRNAs observed in a microarray analysis of cerebellar samples from patients with FXTAS compared to age-matched controls [39].

In contrast to the cerebellar transcriptomics, the pair-wise comparison of peripheral blood gene profiles between FXTAS patients and control donors revealed the predominance of upregulation (12 of 14 miRNAs consistently changed in microarray and next-generation sequencing platforms) [75]. Of these, only miR-27a was also upregulated among the miRNAs derived from extracellular vesicles or lipoprotein complexes [164] of cell-free plasma of our own cohort (see Table A1 Appendix A). This miR-27a was also detected in the serum of individuals affected by age-related macular degeneration [165] and can serve as a potential candidate for further studies as Fragile X-associated biomarkers.

### 2.6. Transcriptional Dysregulation in Fragile X-Associated Syndromes

Despite the intranuclear inclusions not containing *bona fide* transcription factors, the presence of histones and laminA/C that indicate general chromatin disorganization, RNA-binding proteins that may regulate gene expression (as in the case of hnRNPs [166]) and splicing factors that may cause the deregulation of specific variants (as in the case of TRA2A [41]) suggest that transcriptional dysregulation may be important in PM cells. Furthermore, miRNA deregulation may modulate the transcript decay of downstream targets [167]. As we already introduced in the section entitled “Production of FMRpolyG peptides”, there are reports indicating the disruption of the nuclear lamina architecture, a dense fibrillar network that participates in chromatin organization among other functions, which may have a profound impact in transcriptional regulation [168,169]. Such disruption is caused by altered nuclear distribution of laminA/C, observed in immunocytochemistry assays as a loss of the ring-like pattern in the inner nuclear membrane in cultured skin fibroblasts from PM carriers, both symptomatic and asymptomatic for FXTAS [170]. This observation was concomitant with the upregulation of the stress-related components CRYAB, HSP27 and HSP70 (that, together with laminA/C, were also present in FXTAS inclusions [36]). Of note, the inducible expression of a reporter gene bearing the 5′UTR of the *FMR1* bearing long CGG repeats in neuroblastoma-derived cell lines is sufficient to compromise cell viability at the same time disrupting the laminA/C architecture [171,172]. Another observation in favour of the importance of altered patterns of gene expression in PM carriers comes from the global reduction of 5-hydroxymethylcytosine (5hmC), considered as an intermediate state towards DNA demethylation [173,174], in the cerebellum of mouse models for FXTAS that partially affected genes involved in neuronal function and brain development [175].

**Figure A2:**
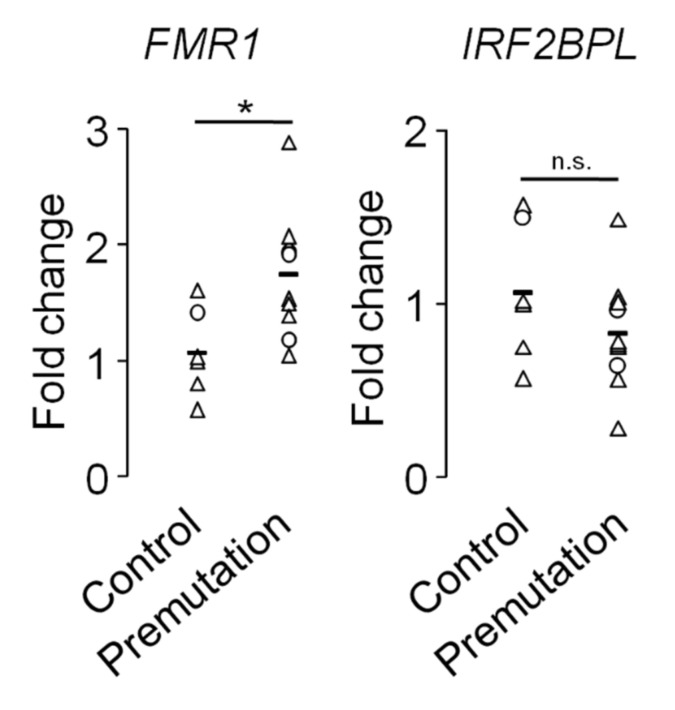
Relative expression of *FMR1* and *IRF2BPL* genes in whole blood of controls and premutation donors. Triangles (Δ), female samples; circles (O), male samples. Of the four males of our cohort, only three were available for these assays. The mean is indicated (horizontal bar). *, *p* < 0.05, Mann-Whitney U-test. n.s., not significant.

A microarray analysis revealed that differential gene expression is observable between FXTAS patients and controls at the peripheral blood level. The results included the deregulation of the following genes: those encoding for respiratory chain subunits and superoxide dismutase 1 (SOD1), mTOR signalling pathway components, chromatin remodelling and epigenetic modulators, related to cell death and, interestingly, those involved in other neurodegenerative conditions, in the case of *ATXN3*, *ATXN7*, *APP* and *TARDP* [76]. Some of these genes were also altered in the brains of mouse models, suggesting that an undetermined component of the PM-associated transcriptional dysregulation is shared between central and peripheral cells. Of note, such direct correlation is difficult to observe in other neurodegenerative disorders and requires additional analytical approaches [176]. Importantly, FXTAS patients showed the downregulation of Interferon Regulatory Factor 2 Binding Protein-like (*IRF2BPL*)/Early at Puberty 1 (*EAP1*), firstly described as a neuronal transcriptional regulator of female reproductive function [177] and later associated with the neurodevelopmental epileptic encephalopathy NEDAMSS (neurodevelopmental disorder with regression, abnormal movements, loss of speech, and seizures, OMIM #618088) [178,179] that was also exacerbated in FXPOI patients compared to asymptomatic PM women [76]. This gene provides an example of a putative biomarker that can be useful for FXTAS, FXPOI and other forms of PM-associated disorders. However, downregulation of this gene was not statistically significant in our cohort (Figure A2 Appendix A), exemplifying the difficulties in the validation of peripheral biomarkers.

The same laboratory also reported the gene expression profile of peripheral blood from PM women with or without FXPOI [180], where differential expression compared to control donors were apparent inconsistent with the previous transcriptomics analysis in FXTAS patients [76], probably indicating that tissue-specific phenomena and normal X-chromosome inactivation may imprint distinct transcriptional signatures in blood cells. Actually, the authors were not able to identify any significant differentially expressed gene (e.g., *IRF2BPL*) between conditions after *P*-value adjustment, although they proposed that pathways related to cell cycle, apoptosis, programmed cell death and survival may be altered in PM carriers with FXPOI when considering the functions associated with the genes showing trends for alteration [180]. Examination of larger cohorts will determine the transcriptional signatures linked to PM and whether these signatures share common components among Fragile X-associated syndromes.

### 2.7. The GABAergic Dysfuntion in Premutation Carriers

GABA is the main inhibitory neurotransmitter in the brain, which deficits have been associated with the cognitive dysfunctions dealing with attention, learning, memory, planning and others that are frequent in neurological disorders including FXS [70,181,182,183]. In the PM condition, there is increasing evidence of impairment in GABA_A_ receptor-mediated transmission. A study using non-invasive brain stimulation protocols in a small cohort of PM females indicated reduced GABA_A_-mediated activity inhibition [184]. In mutant mice, mRNA expression was altered for several subunits encoding GABA_A_ receptors and other components associated with the GABA signalling pathway: downregulation in the cortex and to a lesser extent in the cerebellum of *Fmr1*-null mutant mice [185,186,187] and upregulation in the cerebellum but not in the cortex in mice carrying the expanded CGG repeats in the PM range, despite *Fmr1* mRNA upregulation in both brain areas [187]. Cultured hippocampal neurons from PM mice exhibited altered patterns of electrical spontaneous activity as measured in multielectrode arrays (MEA) that consisted of clustered burst firing interspaced with brief periods of inactivity, resulting in higher spike frequency and longer mean burst duration than wild-type neurons; the patterns of Ca2+ oscillations were also altered in a similar fashion [188]. This behaviour was linked to an imbalance in the excitatory mGluR1/5 and inhibitory GABA_A_-dependent signalling pathways, paralleled by a decrease in the levels of vesicular GABA and Glu transporters. Either inhibition of mGluR1/5 receptors by selective antagonists or enhancement of the GABA_A_ receptor activity by the allosteric modulator allopregnanolone restored the spontaneous firing activity of PM neurons, meanwhile opposite pharmacological manipulations reproduced the clustered burst firing in wild-type neurons [188].

In the case of *FMR1* PM carriers, individuals of both sexes exhibited a differential metabolomic profile in plasma compared to controls that indicated a slow tricarboxylic acid cycle activity, incipient neuronal degeneration and degradation of neuromodulatory fatty acid amides that may be interrelated [154]. Regarding GABAergic transmission, this profile included the aforementioned metabolic intermediates of the Krebs’ cycle (e.g., citrate, aconitate and isocitrate) that might link mitochondrial dysfunction with altered biogenesis of Krebs’ cycle-associated neurotransmitters Glu, Asp and GABA, and the bioactive fatty acid oleamide that can modulate multiple neuronal receptors including GABAergic ones [189,190]. Restoration of GABA function by allopregnallone treatment can improve cognition and executive function, working and episodic memories and anxiety in six FXTAS patients [191], leaving a differential metabolomics signature between pre- and post-treatment that included metabolites related to oxidative stress, mitochondrial function and GABA receptor activity [192], demonstrating the feasibility to find biomarkers related to Fragile X-associated dysfunctions that can be followed up in response to specific treatments.

### 2.8. Telomere Shortening in Premutation Carriers

Telomere lengths consist of TTAGGG repeats in tandem that are reduced by multiple conditions including cellular aging, cellular senescence and apoptosis, cancer processes, heart and lung conditions, Alzheimer’s disease, etc. [193,194,195,196,197,198,199,200]. Shorter telomeres have been described in PBMCs of PM males compared to controls or FM carriers, regardless of FXTAS or dementia diagnosis [201,202,203]. In PM female carriers, this shortening was also present, although the difference related to control donors was more evident with a FXPOI diagnosis in young individuals [204]. This observation is tentative to be generalized to any type of POI, as another study revealed that peripheral leukocytes from women suffering idiopathic POI (i.e., showing amenorrhea and high levels of FSH before 40 years of age, but without any known genetic and environmental cause of chromosome instability) were significantly shorter than controls [205]. Currently, no mechanistic explanation has been offered to explain telomere shortening in peripheral leukocytes except for a generic acceleration of cellular senescence, although other factors can contribute such as the postmenopausal treatment of POI patients with estrogen [206].

## 3. Conclusions

Intensive studies aimed at identifying appropriate biomarkers to predict and/or monitor the complex diversity of symptomatology outcomes of the adult Fragile X-associated syndromes have identified interesting correlations between the most affected organs, mainly focused on neural and reproductive tissues. The expanding catalogue of clinical signs that also indicate affectation in other organs (e.g., heart) demands an additional effort to establish new correlations. We already have a large variety of peripheral blood measurements (e.g., levels for *FMR1* mRNA and *FMR1*-associated lncRNAs, circulating metabolites and ROS, endocrine hormones, etc.) that can be extended to novel candidates thanks to the application of proteomics, metabolomics and transcriptomics. However, it seems that we need to further assess these biomarkers in tailored longitudinal studies to precisely establish consistent correlations with the development and progression of specific outcomes in PM carriers. Furthermore, few studies have gone beyond the correlative evidence by addressing the connection between the organ dysfunction and the peripheral alterations from a mechanistic point of view. Additionally, we should keep in mind that we implicitly assume that the PM and its effects are uniform across the organism and the peripheral cells behave as nearly carbon-copies of the inaccessible cells of interest. Enhancing our comprehension regarding the molecular mechanisms of PM toxicity is key to understand the alterations occurring at the level of both individual cells and cellular networks, and to provide improved tools for the clinical management of the PM carriers in a personalized manner.

## Figures and Tables

**Table 1:** Studies conducted in PM carriers searching for correlation between brain imaging measurements and *FMR1*-derived parameters: number of CGG repeats, *FMR1* mRNA levels and FMRP expression.

PM Carriers	Correlated Signs	*FMR1* Output	Reference
Males	Less voxel density in grey and white matter of brain areas, including cerebellum, brainstem and others	CGG repeats (no correlation with mRNA)	[92]
	Diminished grey matter of amygdala and hippocampal complex, left thalamus and brainstem	% FMRP^+^-lymphocytes (no correlation with mRNA)	
Males	Reduced left hippocampal activation during recall task, correlated with psychiatric assessment in the absence of hippocampal volume change	mRNA (no correlation with CGG repeats)	[93]
Males without FXTAS	Reduced activity of right ventral inferior frontal cortex in verbal working memory in both carriers	mRNA	[94]
Males	Decreased parahippocampal activation in working memory task	Blood FMRP levels	[95]
Females without FXTAS	Activity of right dorsolateral prefrontal cortex in correct encoded trials	mRNA	[96]
	Decreased fronto-parietal activity in a magnitude estimation task	CGG repeats (no correlation with mRNA)	
Males with/without FXTAS	White matter structural connectivity of thesuperior cerebellar peduncles in both carriers	CGG repeats and mRNA	[97]
Males	Cerebellar volume	CGG repeats	[98]
	Defective anticipatory postural responses during stepping	mRNA and CGG repeats	
Males without FXTAS	Motor dysfunction: tremor, balance and brain activation during random finger tapping	(no correlation with mRNA)	[99]

## Data Availability

De novo datasets generated and analyzed during the current study are available in the GEO repository under the Accession Number GSE181018.

## Appendix A

Volunteers consisted of a total of six controls (five females and one male) and 11 PM carriers (eight females and three males) with ages expressed as median (interquartile range) of 42.5 (5.5) and 44 (21.8), respectively (*P*-value = 0.88, Mann Whitney U-test). PM carriers were recruited from family pedigrees with diagnosed cases of FXS, with number of CGG repeats of 103 (11.5).

Peripheral blood from these donors was collected in Tempus Blood RNA tubes (Applied Biosystems, Thermo Fisher, Madrid, Spain) and in EDTA-K2 Blood tubes (BD Vacutainer, Madrid, Spain) in parallel. From the Tempus tubes, total blood RNA was purified with the Preserved Blood RNA Purification Kit I (Norgen Biotek, Thorold, ON, Canada) (including on-column DNAse I digestion) and reverse transcribed into cDNA using the RevertAid First-Strand cDNA Synthesis kit (Fermentas, Thermo Fisher, Madrid, Spain), following manufacturers’ instructions. qPCR was performed in the Rotor-Gene 6000 Detection System (Corbett) using PyroTaq EvaGreen qPCR Mix Plus (Cmb-Bioline, Madrid, Spain). The PCR cycling conditions were as follows: 95 °C for 15 min and 40 cycles of 95 °C for 15 s, 60 °C for 20 s, 72 °C for 20 s. Each independent sample was normalized using *GAPDH* and *ACTB* and the relative quantitative values were calculated according to the 2^−ΔΔCT^ method.

From the EDTA-K2 tubes, plasma was obtained after centrifugation at 3000× *g* 4 °C for 20 min and was kept at −80 °C until experimentation. Thirty µL of plasma was used to determine protein carbonylation as marker of oxidative stress according to the method described previously [207]. Results were normalized with total protein concentration in plasma as determined using the Pierce BCA Protein Assay Kit protocol (Thermo Fisher, Madrid, Spain). Carbonyl content was expressed as nanomoles / mg plasma protein using a molar extinction coefficient of 0.022 mM^−1^cm^−1^. Two hundred µL of plasma was used to isolate circulating small RNA using the miRNeasy Serum/Plasma Kit (Qiagen, Hilden, Germany) following manufacturer’s instructions.

Small RNA was sent to the Genomics Unit of Centro Pfizer-Universidad de Granada-Junta de Andalucía de Genómica e Investigación Oncológica (GENYO) for library preparation (TruSeq Small RNA Library Preparation Kit, Illumina, San Diego, CA, USA) and single-end sequencing (NextSeq, Illumina), following Illumina’s recommendations. After adapter removal using “Cutadapt” [208], reads were aligned to the human genome (hg38 build) using “Bowtie2” (http://bowtie-bio.sourceforge.net/bowtie2/index.shtml, accessed on 02-08-2021) at the Bioinformatics Unit of GENYO. The following Bioconductor-based software packages were used for conversion of BAM files (“RSamtools”) and gene annotation (“GenomicFeatures” and “GenomicAlignments”) using the Ensembl annotation package “Db.Hsapiens.v86” after filtering the “miRNA” gene biotype. Transcriptomics differential expression was determined between control donors and PM carriers by using DESeq2 software [209]. Results (adjusted *p*-value < 0.1) are shown in Table A1 of this Appendix and were compared to the microarray and RNA-seq data obtained from whole blood profiling reported in Table S3 of the original publication [75].

**Table A1:** Differential expression of miRNAs in the plasma of premutation vs. control donors. Potential common genes with a previous report comparing the whole blood profiles from FXTAS patients vs. control donors [75] are indicated as * (upregulation) or ** (downregulation).

miRNA	Ensembl ID	Fold Change (log_2_)	Adj *p*-Value
MIR375	ENSG00000198973	−2.58	5.04 × 10^−5^
MIR483**	ENSG00000207805	−2.72	1.06 × 10^−2^
MIR4664	ENSG00000265660	−4.30	2.58 × 10^−2^
MIR10B	ENSG00000207744	−1.47	3.72 × 10^−2^
MIR7159	ENSG00000276824	−2.95	3.72 × 10^−2^
MIR125B2	ENSG00000207863	−1.67	4.82 × 10^−2^
MIR6730	ENSG00000276830	−4.16	6.60 × 10^−2^
	ENSG00000280773	−5.51	8.17 × 10^−2^
MIR501	ENSG00000211538	−2.10	9.14 × 10^−2^
MIR885**	ENSG00000216135	−2.63	9.99 × 10^−2^
MIR27A*	ENSG00000207808	1.29	9.98 × 10^−2^
MIR26A1	ENSG00000199075	0.75	9.72 × 10^−2^
MIR381	ENSG00000199020	4.10	8.49 × 10^−2^
MIR655	ENSG00000207646	5.02	8.49 × 10^−2^
MIR377	ENSG00000199015	4.64	6.60 × 10^−2^
MIR654	ENSG00000207934	5.00	6.60 × 10^−2^
MIR376C	ENSG00000283279	4.77	6.60 × 10^−2^
MIR494	ENSG00000194717	2.52	5.56 × 10^−2^
MIR889	ENSG00000216099	5.12	6.72 × 10^−3^
MIR134	ENSG00000207993	5.72	5.14 × 10^−3^
MIR331	ENSG00000199172	5.19	3.57 × 10^−3^
MIR766	ENSG00000211578	5.53	8.50 × 10^−4^
MIR877	ENSG00000216101	5.11	1.26 × 10^−4^
MIR495	ENSG00000207743	5.18	5.74 × 10^−5^
